# Prevalence Rates of the Incubus Phenomenon: A Systematic Review and Meta-Analysis

**DOI:** 10.3389/fpsyt.2017.00253

**Published:** 2017-11-24

**Authors:** Marc L. Molendijk, Harriët Montagne, Ouarda Bouachmir, Zeynep Alper, Jan-Pieter Bervoets, Jan Dirk Blom

**Affiliations:** ^1^Faculty of Social and Behavioural Sciences, Leiden University, Leiden, Netherlands; ^2^Leiden Institute for Brain and Cognition, Leiden University Medical Center, Leiden, Netherlands; ^3^Parnassia Psychiatric Institute, The Hague, Netherlands; ^4^Department of Psychiatry, University of Groningen, Groningen, Netherlands

**Keywords:** compound hallucination, parasomnia, rapid eye movement sleep, sleep paralysis, sleep-wake disorder

## Abstract

**Background:**

The incubus phenomenon is a paroxysmal sleep-related disorder characterized by compound hallucinations experienced during brief phases of (apparent) wakefulness. The condition has an almost stereotypical presentation, characterized by a hallucinated being that exerts pressure on the thorax, meanwhile carrying out aggressive and/or sexual acts. It tends to be accompanied by sleep paralysis, anxiety, vegetative symptoms, and feelings of suffocation. Its prevalence rate is unknown since, in prior analyses, cases of recurrent isolated sleep paralysis with/without an incubus phenomenon have been pooled together. This is unfortunate, since the incubus phenomenon has a much greater clinical relevance than isolated sleep paralysis.

**Methods:**

PubMed, Embase, and PsycINFO were searched for prevalence studies of the incubus phenomenon, and a meta-analysis was performed.

**Results:**

Of the 1,437 unique records, 13 met the inclusion criteria, reporting on 14 (*k*) independent prevalence estimates (total *N* = 6,079). The pooled lifetime prevalence rate of the incubus phenomenon was 0.19 [95% confidence interval (CI) = 0.14–0.25, *k* = 14, *N* = 6,079] with heterogeneous estimates over different samples. In selected samples (e.g., patients with a psychiatric disorder, refugees, and students), prevalence rates were nearly four times higher (0.41, 95% CI = 0.25–0.56, *k* = 4, *n* = 1,275) than in the random samples (0.11, 95% CI = 0.08–0.14, *k* = 10, *n* = 4,804). This difference was significant (*P* < 0.001).

**Conclusion:**

This review and meta-analysis yielded a lifetime prevalence of the incubus phenomenon in the general population of 0.11 and, in selected samples, of 0.41. This is slightly higher than the prevalence rates in previous analyses that included cases of recurrent isolated sleep paralysis without an incubus phenomenon. Based on the condition’s robust clinical presentation and the relatively high prevalence rates, we advocate inclusion of the incubus phenomenon as a diagnostic category in major classifications such as the *International Classification of Diseases and Related Health Problems* and the *Diagnostic and Statistical Manual of Mental Disorders*. Recommendations are also made for clinical practice and future research.

## Introduction

**Figure 1:**
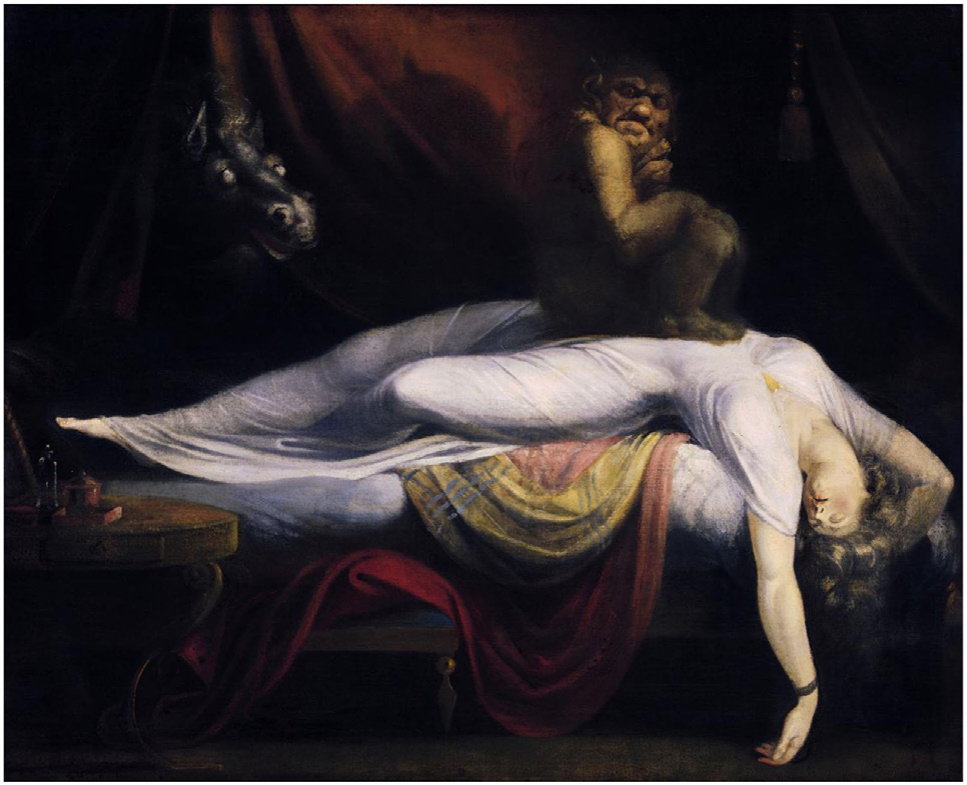
The Nightmare: oil painting by Henry Fuseli (1781).

The incubus phenomenon is a paroxysmal sleep-related disorder, characterized by a feeling of pressure on the chest, while the sleeping individual has the sensation of being awake. Attacks are typically accompanied by sleep paralysis and compound hallucinations involving a creature sitting or lying on the thorax, exerting pressure, and carrying out aggressive and/or sexual acts (Figure 1). The creature may appear in the shape of a human, animal, or metaphysical being, or be of an indeterminate nature. Attacks may occasionally commence with a scream whereas, for the remainder of the time, persons experiencing an attack tend to be mute. Although they may be able to move their eyes, atonia of the striate muscles prevents them from making any other movements. Attacks are usually accompanied by the feeling of a sensed presence and by vegetative symptoms such as piloerection, a cold sweat, tachycardia, hypertension, a feeling of suffocation, and sometimes also sexual arousal. The duration tends to be in the order of seconds to minutes, culminating in a feeling of severe dread and the conviction that one is about to die. Around that time, the sleep paralysis tends to come to an abrupt ending and the hallucinated creature appears to fall or glide from the bed, leaving its victim behind in a state of anxiety and hyperarousal, being unable to go back to sleep out of fear for repetition (1).

The incubus phenomenon is classified as a type of parasomnia and attributed to a dissociation of sleep phases, i.e., a mixture of wakefulness and intrusions of rapid eye movement (REM) sleep-derived hallucinations in which the threat-activated vigilance system plays an important role (2–4). While the experience tends to be highly realistic in nature and the accompanying fear is often described as “off the scale” (5, 6), it is as yet unknown whether the patient’s fear of dying is justified and whether the condition is or is not associated with sudden unexpected death (7). What is known is that it may lead to insomnia, comorbid anxiety disorder, or comorbid delusional disorder and that it should not be confused with (or treated as) a schizophrenia spectrum disorder (8).

### Rationale

Somewhat confusingly, epidemiological studies on the incubus phenomenon are often studies on sleep paralysis in disguise, as if the two were interchangeable phenomena. However, sleep paralysis is a physiological state of atonia that recurs several times during a normal night’s sleep, of which we are completely unaware as long as we do not wake up in the middle of an episode, and/or attempt to move. Epidemiological studies of “recurrent isolated sleep paralysis,” as the condition is officially called by the American Academy of Sleep Medicine (4), have yielded widely varying results, with prevalence rates in the general population ranging from 0.05 in Germany (9) to 0.62 in Canada (10). A meta-analysis by Sharpless and Barber (11) yielded prevalence rates for recurrent isolated sleep paralysis of 0.08 for the general population, 0.28 for students, and 0.35 for psychiatric patients with varying diagnoses. In a different study, an even higher rate was found in narcolepsy patients, i.e., 0.49 (9). In many studies, however, no distinction was made between patients suffering from sleep paralysis with an incubus phenomenon and those without. As a consequence, the prevalence rate of the incubus phenomenon itself remains unknown.

### Objective

The present study aimed to arrive at a reliable estimate of the prevalence rate of the incubus phenomenon in the general population as well as in selected samples, i.e., patients diagnosed with a psychiatric disorder, and otherwise selected groups as described in the literature.

## Methods

### Study Design

We conducted a systematic review and meta-analysis of existing epidemiological studies reporting on the prevalence of the incubus phenomenon.

### Participants, Interventions, Comparators

Records were considered to be eligible when they were published in peer-reviewed journals (including advanced online publications) and reported on the lifetime or point prevalence of the incubus phenomenon. Studies had to be written in English, German, Spanish, or Dutch. Case studies were excluded, as were reviews, meta-analyses, and perspectives that did not contain original data.

### Systematic Review Protocol

The present methodology adhered to the guidelines for the preferred reporting items for systematic reviews and meta-analyses (12). Search procedures, study selection, quality assessment, and data extraction were performed independently by at least two of the authors. Discrepancies were resolved during consensus meetings.

### Search Strategy

A systematic search was made for papers that reported on prevalence rates of the incubus phenomenon. The date of the last search was August 3, 2017. The following string of search terms was used: incubus OR sleep paralysis OR hypnopompic OR hypnagogic. The search was broad because we suspected that studies might exist that reported on the incubus phenomenon only indirectly (e.g., in the context of recurrent isolated sleep paralysis) and thus would fail to mention the incubus phenomenon in the title, abstract or keywords. The digital searches were supplemented by backward searches.

### Data Sources, Studies Sections, and Data Extraction

A search was made in PubMed, Embase, and PsycINFO. From eligible records, data on the following variables were extracted: (i) year of publication, (ii) country in which the study was performed, (iii) demographic and clinical characteristics of the sample, and (iv) the (lifetime or point) prevalence rate of the incubus phenomenon. To assess the methodological quality of the studies included, the Newcastle-Ottawa Scale (cohort version) was used (13), which is the recommended tool for this purpose (14).

### Data Analysis

All analyses were carried out using STATA version 13 (15). Pooled prevalence rates were calculated in a random-effects meta-analysis using the *Metaprop* command. This command applies a double-arcsine transformation to binomial data that allows for confidence intervals (CIs) within admissible values (16). Between-study heterogeneity in outcome was evaluated using the *I*^2^ value (17). To explain potential between-study heterogeneity, analyses were run as a function of whether the prevalence estimate of a particular study was derived from a *random* or *selected* sample. We considered a sample to be *random* when the prevalence of the incubus phenomenon was assessed in a sample not selected with regard to the outcome or features related to it. A sample was considered *selective* when: (i) a specific sample was used (e.g., a sample of persons known to be suffering from sleep paralysis) and (ii) the likelihood of self-selection bias was large and might have yielded a biased sample (e.g., recruitment partly based on the outcome) (18). We aimed to explain the remaining between-study heterogeneity (when present) with the aid of the following variables (without *a priori* hypotheses): mean age and gender distribution of the population, size of the sample, and the methodological quality score of the study. Finally, summary tables were created showing the characteristics of the included studies.

## Results

### Study Selection and Characteristics

**Figure 2:**
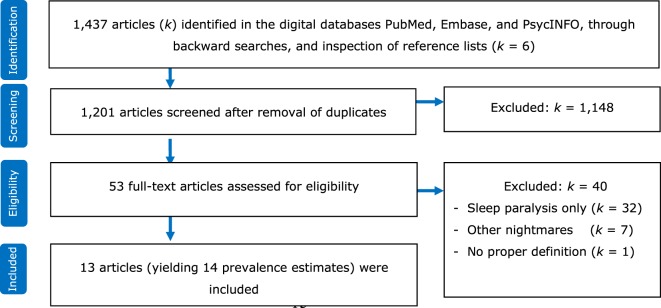
Flowchart for identification, screening, and inclusion of eligible publications.

The literature search yielded 1,437 unique records. After removal of duplications, the titles and abstracts of 1,201 records were reviewed to determine their eligibility. Of these, 53 were considered eligible for full-text assessment. Finally, 13 records met the inclusion criteria. These 13 records reported on 14 (*k*) independent prevalence estimates. Figure 2 is a flow diagram showing the identification, screening, and inclusion of eligible publications. Table 1 lists the included studies by year of publication and alphabetically within a single year. Full references are provided in the reference list.

**Table 1:** Quality assessment of the included studies on the prevalence of the incubus phenomenon.

Study	1	2	3	4	5	6	7	Total
Wing et al. (19)	⊗	N/A	⊘	⊗	⊕	⊕	N/A	0
Spanos et al. (20)	⊗	N/A	⊘	⊗	⊘	⊕	N/A	−1
Fukuda et al. (21)	⊘	N/A	⊘	⊗	⊘	⊕	N/A	0
Cheyne et al. (22)	⊗	N/A	⊘	⊗	⊘	⊕	N/A	−1
Wing et al. (23)	⊕	N/A	⊕	⊗	⊘	⊕	N/A	2
Buzzi and Cirignotta (24)	⊘	N/A	⊘	⊗	⊘	⊘	N/A	−1
Cheyne and Girard (25)	⊕	N/A	⊘	⊗	⊘	⊕	N/A	1
Abrams et al. (26)	⊕	N/A	⊘	⊗	⊘	⊕	N/A	2
Ramsawh et al. (27)	⊕	N/A	⊘	⊗	⊘	⊗	N/A	0
Solomonova et al. (28)	⊗	N/A	⊘	⊗	⊘	⊕	N/A	−1
Jiménez-Genchi et al. (29)	⊕	N/A	⊕	⊗	⊘	⊕	N/A	2
Paradis et al. (30)	⊗	N/A	⊘	⊗	⊘	⊕	N/A	−1
Young et al. (31)	⊗	N/A	⊘	⊗	⊘	⊕	N/A	−1

*⊕ = +1 point; ⊗ = −1 point; Ø = 0 point; N/A, not applicable*.

### Synthesized Findings

The number of participants in the included studies ranged from 72 to 1,798 (mean = 434, sum = 6,079). The average age of the participating individuals was 28 (SD = 16) years. The majority of the included studies showed an overrepresentation of females (average percentage of females = 61, SD = 14). Four studies were performed in Canada (29%), three studies were performed in the USA (22%), two studies were performed in China (14%), one study was performed in Italy (7%), one study was performed in Japan (7%), one study was performed in Mexico (7%), and two studies were performed in >1 country (14%). All studies reported lifetime prevalence rates of the incubus phenomenon. Ten (71%) studies took random samples, whereas the remaining four (29%) studies selectively assessed samples in which the incubus phenomenon was overrepresented compared with the general population (see Methods for the conceptualization of samples into random *versus* selected). For additional information on the included samples, see Tables 1 and 2. Tables 3 and 4 show the items that compose the Newcastle-Ottawa Scale, as well as the item and total methodological scores per included study.

**Table 2:** Characteristics of the included studies.

Study	Prevalence	Measurement	Exclusion	Ethnicity	IP associations
Wing et al. (19)	0.18^a^	GOPQ, lifetime	No criteria	Chinese	None reported
Spanos et al. (20)	0.08^a^	Custom-made, lifetime	No criteria	Unknown	None reported
Fukuda et al. (21)	0.15	Custom-made, lifetime	No criteria	Caucasian	None reported
Fukuda et al. (21)	0.09	Custom-made, lifetime	No criteria	Asian	None reported
Cheyne et al. (22)	0.12	WUSES, lifetime	No criteria	Unknown	Associations with other hallucinatory experiences
Wing et al. (23)	0.09^a^	GOPQ, lifetime	No criteria	Chinese	None reported
Buzzi and Cirignotta (24)	0.13^a^	Custom-made, lifetime	Medical condition	Caucasian	None reported
Cheyne and Girard (25)	0.14	WUSES, lifetime	No criteria	Unknown	Associations with intruder- and vestibular-motor experiences and fear
Abrams et al. (26)	0.40^e^	WUSES, lifetime	No criteria	Mix, 82% Caucasian	Associations with childhood sexual abuse
Ramsawh et al. (27)	0.27^e^	Custom-made, lifetime	No criteria	Mix, 83% Caucasian	None reported
Solomonova et al. (28)	0.65^b^	WUSES, lifetime	See^b^ below	Unknown	Associations with distress
Jiménez-Genchi et al. (29)	0.11^a^	Custom-made, lifetime	No criteria	Mexican	None reported
Paradis et al. (30)	0.04	USEQ, lifetime	No criteria	Mix, 71% Caucasian	None reported
Young et al. (31)	0.31^d^	GOPQ, lifetime	See^c^ below	Miao	None reported

*GOPQ, Ghost Oppression Phenomenon Questionnaire with interview component; USEQ, Unusual Sleep Experiences Questionnaire; WUSES, Waterloo Unusual Sensory Experiences Survey*.

*^a^These estimates include episodes of sleep paralysis with pressure on the chest with or without experiencing difficulties with breathing*.

*^b^This sample selectively recruited participants in online groups concerned with sleep paralysis and related issues; it selected on a correlate of outcome. Hence the prevalence estimate derived in this study is not representative for the general population*.

*^c^This sample is not representative for the general population. It sampled among Hmong immigrants in order to learn about the astonishingly high mortality rate due to the “sudden unexplained death syndrome,” a sleep-related disorder*.

*^d^In this sample, participants were asked whether they experienced “dab tsog,” which in the Hmong culture refers to “a visit from the chest-pressing spirit,” which is equivalent to the Incubus phenomenon and a specific cultural stress regarding issues like this*.

*^e^Recruited through newspaper advertisements, thus, self-selection bias is likely*.

**Table 3:** Items of the Newcastle–Ottawa Scale.

Item	Points		

	Yes	No	Not known
**Selection**			
1. Representativeness of the sample	⊕ representative (random)	⊗ not representative (selected)	⊘ do not know
2. Sample size	This item is not relevant here		
3. Non-respondents	⊕ comparable + high response rate	⊗ not comparable + low response rate	⊘ do not know
4. Ascertainment of exposure	⊕ validated	⊗ not validated/no description	⊘ do not know

**Comparability**			
5. Comparability of subjects	⊕ comparable/controlled	⊗ not comparable/not controlled	⊘ do not know

**Outcome**			
6. Assessment of outcome	⊕⊕ independent/blind linkage ⊕ self-report	⊗ no description	⊘ do not know
7. Statistical test	This item is not relevant here		

*⊕ = +1 point; ⊗ = −1 point; Ø = 0 point*.

**Table 4:** Sample characteristics of the included studies.

Study	*N*	Sample	Percentage females	Mean age (years)	Country
Wing et al. (19)	603	Students	42	21	China
Spanos et al. (20)	1,798	Students	54	20	Canada
Fukuda et al. (21)	86	Students	73	21	Canada
Fukuda et al. (21)	149	Students	41	19	Japan
Cheyne et al. (22)	870	Students	56	20	Canada
Wing et al. (23)	158	General population	58	80	China
Buzzi and Cirignotta (24)	264	Mix	Unknown	Unknown	Italy
Cheyne and Girard (25)	383	Mix	73	31	Mix
Abrams et al. (26)	263	Mix students/general community	73	22	Canada
Ramsawh et al. (27)	72	Mix students/general community	74	25	USA
Solomonova et al. (28)	193	Persons with sleep paralysis, of whom 85% were psychiatric patients	66	32	Canada
Jiménez-Genchi et al. (29)	385	School children/adolescents	67	16	Mexico
Paradis et al. (30)	208	Students	81	22	USA
Young et al. (31)	747	Hmong immigrants	38	40	USA

**Figure 3:**
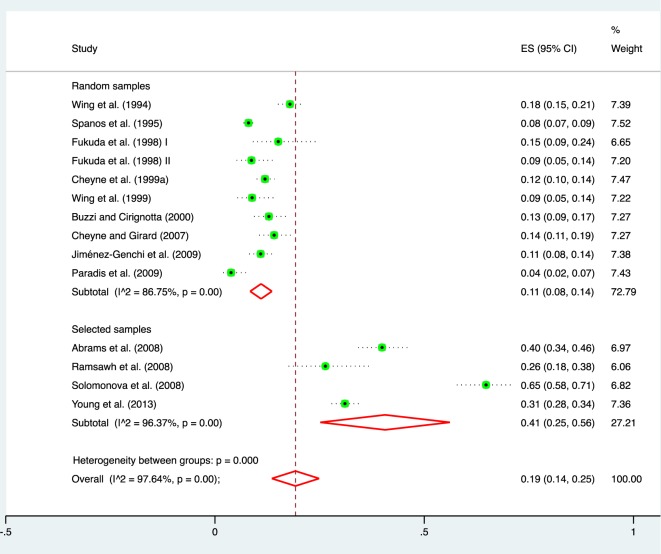
Forest plot for the random-effects model estimating the pooled prevalence of the incubus phenomenon. Note that prevalence estimates are also shown as a function of whether the estimate was derived from a random or a selected sample (e.g., a sample of persons with sleep paralysis).

The pooled lifetime prevalence rate of the incubus phenomenon was 0.19 (95% CI = 0.14–0.25, *k* = 14, *N* = 6,079) (Figure 3). Substantial heterogeneity was found in outcome between the studies (*I*^2^ = 97.60, *Q* = 551.61, *P* ≤ 0.001).

In the selected samples, the pooled prevalence rate of the incubus phenomenon was about four times higher (0.41, 95% CI = 0.25–0.56, *k* = 4, *N* = 1,275) than in the random samples (0.11, 95% CI = 0.08–0.14, *k* = 10, *N* = 4,804); this difference in estimates was significant (*P* < 0.001) (Figure 2). Even when accounting for variance due to selected and unselected samples, significant between-study heterogeneity remained. However, this could not be explained by the variables that were *a priori* defined as potential effect modifiers, i.e., average age (*P* = 0.94), percentage female participants (*P* = 0.82), and methodological quality (*P* = 0.81). The categorical variables “type of population,” “ethnicity,” and “measurement method” could not be related to outcome because they were too diverse (i.e., >4 categories) to reasonably pool them together and test them with sufficient statistical power.

Given that sleep paralysis is reported to have a higher prevalence in student samples relative to non-student samples derived from the general population (11), we also tested for differences in prevalence rates of the incubus phenomenon between these specific groups. The prevalence rates of the incubus phenomenon were somewhat higher in student samples (0.11, 95% CI = 0.08–0.14, *k* = 7, *N* = 3,714) relative to non-student samples (0.08, 95% CI = 0.05–0.15, *k* = 1, *N* = 158); however, this difference was not significant (*P* = 0.55).

### Risk of Bias

A test of publication bias was considered irrelevant, since our main outcome was a straightforward prevalence rate. That said, as noted earlier, we suspect that some of the original studies included in our analysis suffered from selection bias due to self-selection of eligible patients.

## Discussion

### Summary of Main Findings

A systematic review and meta-analysis of published studies was performed to provide (for the first time) an estimate of the prevalence rate of the incubus phenomenon. In the general population, its lifetime prevalence was found to be 0.11 and in selected samples 0.41.

### Historical and Cultural Perspective

The incubus phenomenon has been known since Antiquity (32) and belongs to a select number of neuropsychiatric syndromes that have shown a remarkably stable clinical presentation over time (33). Moreover, it appears to be a universal phenomenon, in the sense that (as far as we know) it does not miss out any historical or contemporary population group. As a consequence, each and every culture has had to come to terms with it. Throughout the ages, this has resulted in numerous explanatory models, ranging from the Greek *Ephialtes* (“he who jumps upon”) (34), and the Japanese *kanashibari* (“bound by metal”) (35), to the Caribbean *kokm*a (the spirit of a deceased new-born) (10), the Mexican *me subió el muerto* (“the dead body climbed upon me”) (29), and the contemporary Western notion of *space alien abduction* (36). As these varying explanatory models appear to be grafted on a rather stereotypic set of clinical phenomena, Cheyne et al. (22) advanced their experiential source hypothesis, which holds that the neurobiological underpinnings of the incubus phenomenon are hard-wired in the brain, while the phenomenological features of (and meaning attributed to) the hallucinated creature are steeped in cultural values, thus yielding a Chinese *yan* (ghost) (19) for each Cambodian *khmaoch sângkât* (ghost of a person killed by the Khmer Rouge) (37, 38) and a Moroccan *boratat* for each Turkish *karabasan* (both meaning incubus) (8).

This brief cultural-historical excursion demonstrates that, in an experiential sense, the incubus phenomenon has always been considered something extraordinary, even though (in an epidemiological sense) it is relatively common and ordinary (as shown here). With a risk of 0.11 for individuals in the general population to experience it at least once during their lives and of 0.41 for selected samples, one would expect it to be a well-known topic, broadly discussed by parents in front of their children, by teachers in front of their pupils, by those with first-hand experience at the end of the bar, if needs be, but (hopefully) first and foremost by teaching staff at universities in front of students of medicine and psychology. However, this is not the case. Instead, the incubus phenomenon is the staple of a fringe group of writers and filmmakers, mostly in the horror genre (39). For the rest, apart from Japanese pop culture with its numerous references to *kanashibari* (40), and a rural population in Canada where the inhabitants are so well-acquainted with the incubus phenomenon that they perform practical jokes to make one another believe that they are having an attack (10), we are still dealing here with a topic that in most societies is hardly known to the greater public and not much better known to many health professionals (41).

This is at least partly due to the fact that the incubus phenomenon does not feature in the *International Classification of Diseases and Related Health Problems* (ICD)-10 (42), which only allows for coding as a Parasomnia Usually Associated With REM Sleep, and from the *Diagnostic and Statistical Manual of Mental Disorders* (*DSM*)-5 (43), which only allows for coding as an Other Specified Sleep-Wake Disorder or an Unspecified Sleep-Wake Disorder. That the incubus phenomenon has a tendency to stay out of mainstream conversations, might be due to the embarrassment that “victims” may experience (notably in the context of sexually charged attacks), to fear of stigmatization or to fear of repetition, as in the case of Muslim patients who dread the wrath of *jinn* when they talk about these metaphysical beings with others (44). It may also be due to the fact that most instances of the incubus phenomenon are non-recurring (45) and that they rarely constitute prodromal symptoms of worse things to come.

That said, recurring attacks may occur. For sleep paralysis, an increased risk is primarily associated with narcolepsy and other sleep disorders (9, 46), alcohol intoxication (47), posttraumatic stress disorder (38), anxiety disorders (48), exploding head syndrome (49), sexual abuse (36), stress (46), and physical illness (46). Other factors that may increase the risk for an attack include a supine sleeping position (especially when complicated by apnea due to airway obstruction) (50), an irregular sleeping pattern (51), and amphetamine use (52).

### Consequences for Research and Clinical Practice

The consequences of our findings for future research and clinical practice are fourfold. In the first place, a better dissemination of knowledge regarding the biomedical conceptualization of the incubus phenomenon in the general population might help to prevent (or at least diminish) catastrophic interpretations and, thus, help diminish the number of cases that go on to develop comorbid pathology. Second, state-of-the-art knowledge among health professionals, including knowledge of the condition’s prevalence and the risk factors associated with sleep paralysis, may help improve diagnosis and prevent improper treatment (8, 53). Third, future editions of the major diagnostic classifications, such as the *ICD* and *DSM* systems, could include a specific diagnostic category describing the incubus phenomenon, to allow for proper diagnosis and an improved awareness of the condition. Obviously, such a diagnostic category will need to provide criteria that allow clinicians to differentiate between “harmless” (i.e., non-recurring) instances, which do not require auxiliary investigations and/or treatment, and those that do (similar to the way conditions such as depressive disorder and anxiety disorders are operationalized). Fourth, much work is required to gain better insight into the neurophysiology and neuropsychology of the incubus phenomenon and its possible (although at present unlikely) association with sudden death (7), and to develop evidence-based treatment protocols for recurrent, isolated cases.

### Limitations

The present study has several limitations. Like all meta-analyses, the quality of the outcome of our study stands and falls with the quality of the included studies. Although we followed strict procedures in conformity with the guidelines for systematic reviews and meta-analyses, we had to depend on the works of the original authors. As the prevalence figures reported in some of those studies were substantially higher than others, we cannot exclude the possibility that some of them may have been biased by self-selection of eligible patients. However, we were able to present prevalence rates for selected and random samples separately. We should note here that the random samples we report on were not composed of healthy individuals alone. The studies that we labeled as “random” did not exclude, for instance, psychiatric patients or patients with sleep disorders. As these variables were not assessed in the input studies, the prevalence rates as we report them in “random samples” may indeed be driven by such subsamples. Hence, the prevalence of the incubus phenomenon might be lower in groups of healthy individuals. Finally, due to the strictness of our procedure, the total number of studies to be analyzed was relatively small, at least in comparison with the number of studies on the related topic of sleep paralysis.

### Conclusion

On the basis of this systematic review and meta-analysis of studies reporting on the prevalence of the incubus phenomenon, its lifetime prevalence rate in the general population was found to be 0.11 (95% CI = 0.08–0.14) and 0.41 (95% CI = 0.25–0.56) in selected samples of patients with sleep paralysis and those with varying psychiatric diagnoses, as well as in refugees.

## Author Contributions

MM contributed to the conception and design of the work and to the acquisition and analysis and interpretation of data for the work, drafted and revised the work, gave final approval for the final version to be published, and agreed to be accountable for all aspects of the work in ensuring that questions related to the accuracy or integrity of any part of the work are appropriately investigated and resolved. HM and OB contributed to the interpretation of data for the work, revised the work, gave final approval for the final version to be published, and agreed to be accountable for all aspects of the work in ensuring that questions related to the accuracy or integrity of any part of the work are appropriately investigated and resolved. ZA and J-PB contributed to the acquisition, analysis, and interpretation of data for the work, revised the work, gave final approval for the final version to be published, and agreed to be accountable for all aspects of the work in ensuring that questions related to the accuracy or integrity of any part of the work are appropriately investigated and resolved. JDB contributed to the conception and design of the work and to the analysis and interpretation of data for the work, drafted and revised the work, gave final approval for the final version to be published, and agreed to be accountable for all aspects of the work in ensuring that questions related to the accuracy or integrity of any part of the work are appropriately investigated and resolved.

## Conflict of Interest Statement

The research was conducted in the absence of any commercial or financial relationships that could be construed as a potential conflict of interest.
